# A random walk description of individual animal movement accounting for periods of rest

**DOI:** 10.1098/rsos.160566

**Published:** 2016-11-23

**Authors:** Paulo F. C. Tilles, Sergei V. Petrovskii, Paulo L. Natti

**Affiliations:** 1 Departamento de Matematica, Universidade Federal de Santa Maria, Santa Maria, Brazil; 2 Department of Mathematics, University of Leicester, Leicester, UK; 3 Departamento de Matematica, Universidade Estadual de Londrina, Londrina, Brazil

**Keywords:** dispersal, composite random walk, movement behaviour

## Abstract

Animals do not move all the time but alternate the period of actual movement (foraging) with periods of rest (e.g. eating or sleeping). Although the existence of rest times is widely acknowledged in the literature and has even become a focus of increased attention recently, the theoretical approaches to describe animal movement by calculating the dispersal kernel and/or the mean squared displacement (MSD) rarely take rests into account. In this study, we aim to bridge this gap. We consider a composite stochastic process where the periods of active dispersal or ‘bouts’ (described by a certain baseline probability density function (pdf) of animal dispersal) alternate with periods of immobility. For this process, we derive a general equation that determines the pdf of this composite movement. The equation is analysed in detail in two special but important cases such as the standard Brownian motion described by a Gaussian kernel and the Levy flight described by a Cauchy distribution. For the Brownian motion, we show that in the large-time asymptotics the effect of rests results in a rescaling of the diffusion coefficient. The movement occurs as a subdiffusive transition between the two diffusive asymptotics. Interestingly, the Levy flight case shows similar properties, which indicates a certain universality of our findings.

## 1. Introduction

Dispersal is a common property of all ecological species [1,2]. Understanding its properties is important for many reasons, in particular because dispersal is known to be a primary factor controlling the rate and extent of species’ spatial spread [3–5]. While dispersal of plants occurs mostly due to the seeds’ transport by environmental flows (such as, depending on the species traits, wind or water flows), dispersal of animals mostly happens due to their self-motion. In this paper, we focus on the dispersal of animals.

Dispersal is usually described by the dispersal kernel^1^ which is essentially the probability density to find the animal at a given location (say *x*) after a certain time (say *t*) of its movement away from the point of its release. A comprehensive approach to dispersal is therefore hardly possible without a good understanding of individual animal movement, and indeed the latter has been a focus of considerable attention and controversy over the last two decades [7–12].

A traditional way to consider the Brownian motion as an appropriate model of individual movement [13,14] (with the dispersal kernel having ‘thin’ exponential or even Gaussian tail corresponding to the ordinary diffusion) was eventually challenged by experimental observations that movement of some animals apparently follow a different pattern where the dispersal kernel has a ‘fat’ tail with a lower rate of decay described by a power-law [11,15–17]. The latter is usually associated with the Levy walk [12] or, more generically, with anomalous diffusion [8]. The rate of decay at the dispersal tail can have huge implications as it is believed to ultimately define the rate of species spread [3]. Theoretical approaches were developed to show that the foraging following the Levy-type movement pattern can optimize the search [18] and hence may arise as a result of evolution [15]. The issue as a whole remains highly controversial though. For some animal species that originally were claimed to perform Levy walks, later analysis re-classified them to the ‘thin-tailed’ Brownian walkers; see [7,11] for albatrosses, [15,19] for mussels, [20,21] for Tenebrio beetles.

Whatever the specific movement pattern is, a straightforward interpretation of a theoretical probability density function (pdf) as an ecologically meaningful dispersal kernel may be misleading because of the existence of different time scales. Standard approaches of statistical mechanics to calculate the propagator (dating back to the original work by Einstein [22]) usually consider a particle performing either uninterrupted movement or movement occasionally interrupted by short stops. Importantly, the duration of these short stops is on the same time scale as the duration of the short steps (e.g. between turning points in the so-called velocity model [6]) along the movement path. The combination of short steps and short stops defines the movement to which we, for convenience, will refer as continuing or uninterrupted. Also in biology, theoretical studies on animal movement aiming to calculate the dispersal kernel and/or the rate of animal spread (e.g. the mean squared displacement (MSD) as a function of time) usually consider an animal performing continuing non-stop movement [14,23–27]. However, this approach is only valid when animal movement is considered over a relatively short time. Indeed, if considered on a larger time scale, the animal would normally alternate long periods of continuing movement (‘bouts’) with long periods of rest [28,29]. It remains unclear how the properties of the dispersal kernel calculated for the uninterrupted movement can change as a result of upscaling when the animal movement is considered over a longer time (or at a larger spatial scale) that includes periods of rest. The goal of this paper is to bridge this gap. We are particularly concerned with the following questions:

— how the presence of rest times can affect the baseline dispersal kernel of uninterrupted movement; in particular, whether a thin-tailed kernel may become fat-tailed and vice versa;
— how the rate of spread (e.g. the dependence of the MSD on time) can be modified.

This paper is organized as follows. In §2, we introduce a model to describe the composite movement consisting of periods of uninterrupted movement and periods of rest and provide a semi-intuitive analysis of its properties. In §3, we perform a rigorous analytical study of the composite movement to obtain a general equation for the corresponding pdf. The mathematical analysis is illustrated and complemented with simulation results. In §4, our mathematical analysis of the movement is extended to address the rate of convergence of the pdf to its large-time asymptotics. Finally, in §5 we discuss the implications of our findings.

## 2. Composite movement: semi-intuitive analysis

We consider an idealized situation of animal foraging in a uniform and stationary environment. Although the assumption of uniformity is likely to be inadequate if the movement is considered in a natural environment on large spatial and temporal scales, arguably it should work well at small and intermediate scales (e.g. imaging a bumble-bee or a small rodent foraging in a large meadow or crop field), and it certainly works well in the case of a laboratory environment. We neglect the size of the animal compared with the typical travel distances, so that its location in space is given by a single point, e.g. ***x***=(*x*,*y*).

Let ρ(x,Dt) be the pdf of the animal position at a time *t*≥0 in the baseline case of ‘uninterrupted’ movement (i.e. without long periods of rest, see §1), where *D* is the distribution parameter; in particular, in the case of diffusion, *D* is the diffusion coefficient.

Let us now consider the situation where the movement stops suddenly at *t*=*τ*_1_, so that the animal is at rest for any *t*>*τ*_1_. Compared with the uninterrupted movement, this is a different stochastic process and it is described by its own pdf ${\hat{\rho }}_{1}(x,Dt)$ which is related to *ρ* as follows:

2.1 $${\hat{\rho }}_{1}(x,Dt)=\rho (x,Dt)\;\text{for}\;0\leq t\leq \tau_{1},\;{\hat{\rho }}_{1}(x,Dt)=\rho (x,D\tau_{1})\;\text{for}\;t\geq \tau_{1},$$

as time after *t*=*τ*_1_ does not contribute to the pdf, because the position of the animal does not change for *t*>*τ*_1_. In the case that *τ*_1_ is a fixed parameter, equations (2.1) provide the full description of the movement (assuming that the baseline pdf ρ(x,Dt) is known). In the case that *τ*_1_ is a random variable, equations (2.1) give the description only for one particular realization of the process and should be averaged over all possible values of *τ*_1_ (in the manner shown in the next section).

Consider now a sequence of several bouts, *τ*_1_,…,*τ*_*n*_, and the corresponding rests separating them, *ξ*_1_,…,*ξ*_*n*−1_. Consider the moment *t*_*n*_ corresponding to the end of the last bout in this sequence, that is

2.2 $$t_{n}=\sum_{k=1}^{n-1}(\tau_{k}+\xi_{k})+\tau_{n}.$$

Using the same arguments as above, it is readily seen that the pdf of the animal position at time *t*_*n*_ is

2.3 $${\hat{\rho }}_{n}(x,Dt_{n})=\rho (x,DT),$$

where *T*=*τ*_1_+⋯+*τ*_*n*_ is the total duration of the actual movement, i.e. total time excluding periods of rest. Moreover,

2.4 $${\hat{\rho }}_{n}(x,Dt)=\rho (x,DT)\;\text{for any}\;t>t_{n}.$$

As an instructive example, let us consider the case where the baseline case of uninterrupted movement is described by a Gaussian dispersal kernel. We then observe that

2.5 $${\hat{\rho }}_{G}(x,Dt_{n})=\rho_{G}(x,DT)=\frac{e^{-x^{2}/DT}}{\sqrt{\pi DT}}=\frac{e^{-x^{2}/D_{*}t_{n}}}{\sqrt{\pi D_{*}t_{n}}}=\rho_{G}(x,D_{*}t_{n}),$$

where

2.6 $$D_{*}=\frac{T}{t_{n}}D.$$

Therefore, for the stochastic process defined by a given sequence of bouts and rests, the pdf of the animal position at time *t*_*n*_ coincides with the pdf of the uninterrupted movement up to the rescaling of the diffusion coefficient.

In any realistic animal movement, the durations of bouts and rests are of course not fixed. Instead, they can be regarded as random variables and described accordingly by their probability density functions, say *ϕ*(*τ*,*λ*) and *ψ*(*ξ*,*ω*), where *λ*^−1^ and *ω*^−1^ are the average duration of bouts and rests, respectively. Correspondingly, expression (2.5) is not the actual probability density function of this composite movement but the conditional one, say $\rho (x,Dt\;|\;\{\tau_{k}\},\{\xi_{k}\})$, i.e. the pdf conditioned by the given sequence of bouts and rests. This conditional pdf then must be averaged over all possible sequences {*τ*_*k*_} and {*ξ*_*k*_} in order to obtain the averaged or expected pdf, which we denote as ⟨ρ(x,Dt)⟩. It is this expected pdf that gives a global description of the movement process taking into account details of the given stochastic process across different time scales.

For a small or intermediate *t*_*n*_, the averaging is likely to turn the simple rescaling relation (2.6) into something more complicated. However, when time becomes large, i.e. when *t*_*n*_≫*λ*^−1^+*ω*^−1^, the movement includes many bouts and rests of various duration. Intuitively, this is equivalent to averaging over different stochastic realizations. One therefore can expect that (2.6) is true asymptotically, i.e. for sufficiently large time. In the next section, these heuristic arguments will be confirmed by a rigorous analysis.

## 3. Rigorous analysis and results

Our analysis is based on the following two non-restrictive assumptions:

— The bout and rest durations are statistically independent random variables fully determined by their probability density functions, i.e. by *ϕ* and *ψ*, respectively;
— Bouts and rests do not present either dependence or influence on other variables of the movement, in particular on the functional form of the baseline pdf ρ(x,Dt).

It does not seem possible to perform the analysis in the case of unspecified pdfs of bouts and rests duration, therefore we have to decide about appropriate functions *ϕ* and *ψ*. Based on substantial experimental evidence [29–31], a suitable choice for the probability distribution of bouts is the exponential distribution

3.1 $$\phi (\tau ,\lambda )=\lambda \;e^{-\lambda \tau }.$$

The situation with the duration of rests is less straightforward. A standard assumption often made in the theoretical literature (sometimes implicitly, see section 2.3.2 in [31] for a discussion of this issue) is that the distribution of the rest times is exponential too

3.2 $$\psi (\xi ,\omega )=\omega \;e^{-\omega \xi }.$$

Recently, there has been growing empirical evidence that, at least in some situations, the distribution of rest times can significantly deviate from the exponential, in particular to exhibit fat tails, cf. [29]. For the sake of analytical tractability, in this section we stick to the standard choice (3.2); possible modification of our results in the case that *ψ* has a fatter tail (e.g. being described by a power law) will be briefly discussed in §5.

Once the statistical properties of bouts and rests are thus defined, our approach consists of computing the probability of having *k* bouts within the observation time *t*, and then gathering the contributions of all possible number of bouts, which goes from one to infinity, to compose the *expected* quantity ⟨ρ(x,Dt)⟩. To proper illustrate the procedure, let us first consider in detail the contribution from a realization with only one bout. If we assume the composite movement to always start with a bout, then at the observation time *t* there are two situations that must be considered: one in which a bout started at *t*=0 is still going and the other in which the bout period has stopped at some time *t*^′^<*t*. In the former case, the dispersal process is active during the whole interval [0,*t*], so its contribution should be the exact function ρ(x,Dt) conditioned on the probability of observing a bout of duration longer than *t*, i.e.

3.3 $$\Delta_{1}^{\text{(b)}}(x,t)=\int_{t}^{\infty }d\tau \phi (\tau ,\lambda )\rho (x,Dt)=e^{-\lambda t}\rho (x,Dt).$$

For the latter case, it is necessary to consider that any realization with a bout of *τ*<*t* will contribute, and that the following (observed) rest period must have started at some time *t*−*τ* and still be going on at the observation moment *t*. When setting up the general expression of this case, we take into account that the functional contribution at *t* can depend only on the bout duration *τ*, and that the constraints on the activity periods should appear in the limits of integration. Using this reasoning, one can readily write down the contribution of this case as

3.4 $$\Delta_{1}^{\text{(r)}}(x,t)=\int_{0}^{t}d\tau \phi (\tau ,\lambda )\int_{t-\tau }^{\infty }d\xi \psi (\xi ,\omega )\rho (x,D\tau )=\lambda \;e^{-\omega t}\int_{0}^{t}d\tau \;e^{(\omega -\lambda )\tau }\rho (x,D\tau ).$$

From these two simple cases, we can extend the formalism onto the general case in which an arbitrary number *k* of bout periods are obtained from the statistical ensemble. Again there are two slightly different situations, so let us first analyse the case in which the *k*th bout period is still ongoing at the observation time *t*. Prior to *t*, there must be exactly *k*−1 bout periods and *k*−1 rest periods, with a total duration no larger than *t*. But as the functional contribution ρ(x,⋅) in *t* does not depend on the specific order in which the periods occur, but rather on the total amount of time spent in bouts, the periods can be joined into two separate intervals with durations *τ* and *ξ*, corresponding to the total duration of bout and rest activities, respectively. As each period is obtained from the sum of *k*−1 exponentially distributed random variables, the resulting description of the total duration is given by a Gamma distribution. Hence, the probabilities of observing a total duration *τ* of bouts and *ξ* of rests obtained from *k* distinct periods are given, respectively, as

3.5 $$\phi_{k}(\tau ,\lambda )=\frac{\lambda^{k}\tau^{k-1}\;e^{-\lambda \tau }}{\Gamma (k)}\;\text{and}\;\psi_{k}(\xi ,\omega )=\frac{\omega^{k}\xi^{k-1}\;e^{-\omega \xi }}{\Gamma (k)}.$$

The same approach may be used to obtain the expected value of the function, which consists of assuming the total duration of the *k*−1 periods of bout and rest to be smaller than observation time, *τ*+*ξ*<*t*, and considering the last bout period (*τ*^′^) to still be ongoing at *t*. The only subtlety is that the functional contribution must depend on the total amount of bout activity, including an unspecified fraction of the last one, but this issue is easily resolved by realizing that the whole bout period must be equal to *t*−*ξ*. So the full contribution of the case in which the *k*th bout period is still ongoing at *t* may be written as

3.6 $$\begin{array}{rl} \Delta_{k}^{\text{(b)}}(x,t) & =\int_{0}^{t}d\xi \psi_{k-1}(\xi ,\omega )\int_{0}^{t-\xi }d\tau \phi_{k-1}(\tau ,\lambda )\int_{t-\xi -\tau }^{\infty }d\tau^{\prime }\phi (\tau^{\prime },\lambda )\rho [x,D(t-\xi )]\\ & =\frac{\lambda^{k-1}\omega^{k-1}}{\Gamma (k)\Gamma (k-1)}\;e^{-\lambda t}\int_{0}^{t}d\xi \;e^{(\lambda -\omega )\xi }\xi^{k-2}{\left(t-\xi \right)}^{k-1}\rho [x,D(t-\xi )]. \end{array}$$

The case in which the *k*th rest period is still ongoing is a very similar, with the only differences being that there must be *k* bout periods prior to *t*, and that the functional contribution may be written directly as dependent on the total bout duration *τ*. Hence this contribution may be written as

3.7 $$\begin{array}{rl} \Delta_{k}^{\text{(r)}}(x,t) & =\int_{0}^{t}d\tau \phi_{k}(\tau ,\lambda )\int_{0}^{t-\tau }d\xi \psi_{k-1}(\xi ,\omega )\int_{t-\xi -\tau }^{\infty }d\xi^{\prime }\psi (\xi^{\prime },\omega )\rho (x,D\tau )\\ & =\frac{\lambda^{k}\omega^{k-1}}{\Gamma {\left(k\right)}^{2}}\;e^{-\omega t}\int_{0}^{t}d\tau \;e^{(\omega -\lambda )\tau }\tau^{k-1}{\left(t-\tau \right)}^{k-1}\rho (x,D\tau ). \end{array}$$

Once the method for computing the contribution from a general ensemble of *k* bout periods has been described, the only thing left to do is to sum all contributions to obtain the expected value ⟨ρ(x,Dt)⟩. As the case for *k*=1 is already accounted for by equations (3.3) and (3.4), the contribution of equations (3.6) and (3.7) should be within a summation that goes from *k*=2 up to infinity

3.8 $$\langle \rho (x,Dt)\rangle =\Delta_{1}^{\text{(b)}}(x,t)+\Delta_{1}^{\text{(r)}}(x,t)+\sum_{k=2}^{\infty }\Delta_{k}^{\text{(b)}}(x,t)+\sum_{k=2}^{\infty }\Delta_{k}^{\text{(r)}}(x,t).$$

Now, if we recall the series representation of the modified Bessel function of the first kind,

3.9 $$I_{n}(x)=\sum_{m=0}^{\infty }\frac{1}{\Gamma (m+1)\Gamma (m+n+1)}{\left(\frac{x}{2}\right)}^{2m+n},$$

then the summations in equation (3.8) easily turn into well-known functions

3.10*a* $$\begin{array}{rl} \sum_{k=2}^{\infty }\Delta_{k}^{\text{(b)}}(x,t) & =e^{-\lambda t}\int_{0}^{t}d\xi \;e^{(\lambda -\omega )\xi }\rho [x,D(t-\xi )]\frac{1}{\xi }\sum_{k=2}^{\infty }\frac{{\left[\lambda \omega \xi (t-\xi )\right]}^{k-1}}{\Gamma (k)\Gamma (k-1)}\\ & =e^{-\lambda t}\int_{0}^{t}d\xi \;e^{(\lambda -\omega )\xi }\rho [x,D(t-\xi )]\sqrt{\frac{\lambda \omega (t-\xi )}{\xi }}I_{-1}(2\sqrt{\lambda \omega \xi (t-\xi )}), \end{array}$$

and

3.10*b* $$\begin{array}{rl} \sum_{k=2}^{\infty }\Delta_{k}^{\text{(r)}}(x,t) & =\lambda \;e^{-\omega t}\int_{0}^{t}d\tau \;e^{(\omega -\lambda )\tau }\rho (x,D\tau )\sum_{k=2}^{\infty }\frac{{\left[\lambda \omega \tau (t-\tau )\right]}^{k-1}}{\Gamma {\left(k\right)}^{2}}\\ & =\lambda \;e^{-\omega t}\int_{0}^{t}d\tau \;e^{(\omega -\lambda )\tau }\rho (x,D\tau )[I_{0}(2\sqrt{\lambda \omega \tau (t-\tau )})-1]. \end{array}$$

When all terms are combined, from (3.8) we obtain an expression that allows for a compact integral representation

3.11 $$\begin{array}{rl} \left\langle \rho (x,Dt)\right\rangle & =e^{-\lambda t}\rho (x,Dt)+\sqrt{\lambda \omega }\;e^{-\lambda t}\int_{0}^{t}d\xi \;e^{(\lambda -\omega )\xi }\sqrt{\frac{t-\xi }{\xi }}I_{-1}\left(2\sqrt{\lambda \omega \xi (t-\xi )}\right)\rho [x,D(t-\xi )]\\ & \;+\lambda \;e^{-\omega t}\int_{0}^{t}d\tau \;e^{(\omega -\lambda )\tau }I_{0}\left(2\sqrt{\lambda \omega \tau (t-\tau )}\right)\rho (x,D\tau ). \end{array}$$

It is possible to further simplify equation (3.11) if we consider a change in the integration variables in order to obtain a single integral representation, which is done by defining *ξ*=*t*(1−*y*) and *τ*=*ty*. We may also consider the following rescaled parameters

3.12 $$\alpha =\frac{\omega }{\lambda },\;\beta =\frac{D}{\lambda },\;\zeta =\lambda t,$$

which reduce the dimensionality of the parameter space. As a result, the expected value of the dispersal process may be written as

3.13 $$\langle \rho (x,\beta \zeta )\rangle =e^{-\zeta }\rho (x,\beta \zeta )+\zeta \;e^{-\alpha \zeta }\int_{0}^{1}dy\;e^{(\alpha -1)\zeta y}\left[\sqrt{\frac{\alpha y}{1-y}}I_{-1}(h)+I_{0}(h)\right]\rho (x,\beta \zeta y),$$

where $h(\zeta ,y)=2\zeta \sqrt{\alpha y(1-y)}$. Equation (3.13) gives the exact behaviour of the expected function at some observation rescaled time *ζ*. However, because of its complexity, the spatial behaviour of the distribution is not readily seen. In order to reveal this behaviour, we have to use some approximations and asymptotic analysis. In the following analysis, it is convenient to consider three time scales: (i) small, (ii) intermediate, and (iii) large.

(i) *Small time asymptotics*. When considering the movement at a very early stage of dispersal, *ζ*≪1 (i.e. *t*≪1/*λ*), the system is predominantly driven by the first bout. Therefore, at the corresponding observation time the process is described by the pdf of non-interrupted movement ρ(x,βζ).

(ii) *Intermediate time asymptotics*. For a larger observation time, i.e. *ζ*∼1/*λ*, the activity switches between bout and rest period become statistically relevant, and their influence on the expected value should be properly addressed. To perform such analysis, it will require an approach that approximates the integrals without obscuring (or locally approximating) the spatial dependence, and at the same time it should be valid for any choice of ρ(x,βζ). One way of accomplishing such feature is to approximate the kernel of the integral around the region where it exhibits its maximum value, in a way that the integrand would become a power series multiplied by the original dispersal function. By considering only the kernel of the integral in equation (3.13), i.e.

**Figure 1.**
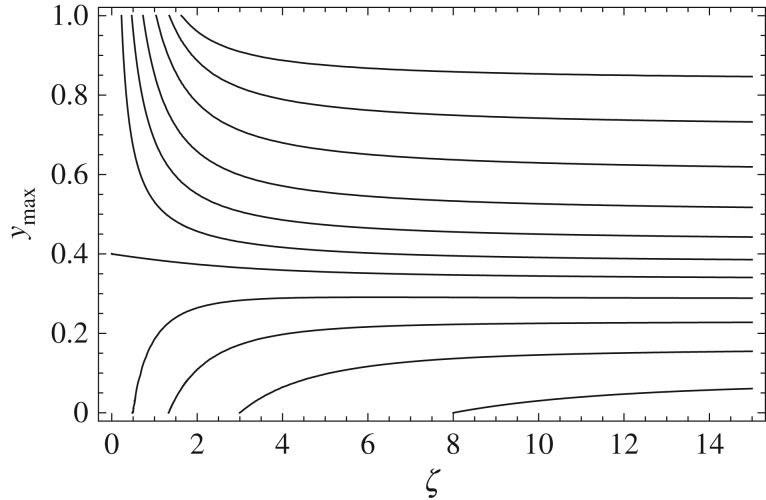
Position $y_{\max}$ of the maximum of the kernel function (3.14) for several values of *α*. On the right, from bottom to top, *α*=0.1,0.2,0.3,0.4,0.5,0.6,0.75,1.0,1.5,2.5 and 5.0. For small values of *α*, the maximum is located at $y_{\max}=0$, as long as *ζ* is also sufficiently small. As *α* increases, the position continuously shifts towards $y_{\max}=1$.

3.14 $$K(\alpha ,\zeta ,y)=\zeta \;e^{-\alpha \zeta }\;e^{(\alpha -1)\zeta y}\left[\sqrt{\frac{\alpha y}{1-y}}I_{-1}(h)+I_{0}(h)\right],$$

then the objective is to obtain a power series in *ζ* expanded around the maximum value of the function defined on the interval [0,1], but the precise location of the maximum depends on both *ζ* and *α*. For *ζ* sufficiently small, the kernel presents its maximum value either at *y*=0 for α≲0.4, or *y*=1 for α≳0.6. In the intermediary range there is a transition in the behaviour, as the kernel function presents a concavity with the maximum, located inside the interval (0,1), being determined by ∂_*y*_*K*(*α*,*ζ*,*y*)|_*y*=*y*_=0. In the general case, it is always possible to determine, at least numerically, the precise position *y*(*α*,*ζ*) of the maximum, so (3.13)–(3.14) turn into the following:

3.15 $$\langle \rho (x,\beta \zeta )\rangle =e^{-\zeta }\rho (x,\beta \zeta )+\sum_{n=0}^{\infty }a_{n}(\alpha ,\zeta )\int_{0}^{1}dy{\left(y-y_{\text{max}}\right)}^{n}\rho (x,\beta \zeta y),$$

where $a_{n}(\alpha ,\zeta )=\partial_{y}^{\left(n\right)}K(\alpha ,\zeta ,y){|}_{y=y_{\text{max}}}$ (auxiliary figure 1 shows the precise location of the maximum in the initial dynamics regime, and the several values of *α* illustrating this transition behaviour indicate the neighbourhood of *y* where the power series should be expanded around). Equation (3.15) is generic and is valid for any dispersal process. However, it is only possible to obtain an explicit expression for ⟨ρ(x,βζ)⟩ when an explicit expression for the baseline pdf ρ(x,βζ) of the non-interrupted movement is available. In §§3.1 and 3.2 below, we consider equation (3.15) for two qualitatively different processes, i.e. for the Brownian motion when ρ(x,⋅) is the normal distribution and for the Levy flight when ρ(x,⋅) is the Cauchy distribution.

(iii) *Large-time asymptotics*. At sufficiently large times, *ζ*≫1, when a relatively large number of bout and rest periods have already taken place, we can use asymptotic methods to approximate equation (3.13). If we consider the asymptotic expansion of the modified Bessel functions,

3.16 $$I_{n}(x)=\frac{e^{x}}{\sqrt{2\pi x}}\left[1-\frac{4n^{2}-1}{8x}+O\left(\frac{1}{x^{2}}\right)\right],$$

then we may consider only the first term of the expansion in order to write

3.17 $$\begin{array}{rl} I(\alpha ,\zeta ) & \equiv \int_{0}^{1}dyK(\alpha ,\zeta ,y)\rho (x,\beta \zeta y)\\ & ≃\frac{\zeta^{1/2}\;e^{-\alpha \zeta }}{2\pi^{1/2}\alpha^{1/4}}\int_{0}^{1}dy\frac{\rho (x,\beta \zeta y)}{{\left[y(1-y)\right]}^{1/4}}\left[1+{\left(\frac{\alpha y}{1-y}\right)}^{1/2}\right]e^{\zeta [(\alpha -1)y+2\sqrt{\alpha y(1-y)}]}. \end{array}$$

In this presented form, the integral (3.17) may be associated with the general expression

3.18 $$I(\zeta )=\int_{0}^{1}dyf(y,\zeta )\;e^{\zeta S(y)},$$

which can be analysed by using the Laplace method to estimate the leading asymptotics for large *ζ*. As the function *f* *e*^*ζS*^ tends to zero for y→0 and y→1 (*f* alone diverges at *y*=1, but the limit ζ→∞ guarantees the convergence), and *S*(*y*) has a unique maximum at

3.19 $$y_{0}=\frac{\alpha }{1+\alpha },$$

the leading order of the integral at large observation times is obtained from

3.20 $$\int_{0}^{1}dy\;f(y,\zeta )\;e^{\zeta S(y)}≃\sqrt{-\frac{2\pi }{\zeta S^{\prime \prime }(y_{0})}}f(y_{0},\zeta )\;e^{\zeta S(y_{0})}.$$

With the aid of the aforementioned approximation methods, we are now ready to further analyse the properties of the dispersal kernel by considering two specific representations for ρ(x,Dt). Having assumed that the environment is uniform and isotropic, we will restrict our analysis to the one-dimensional case. We will consider two qualitatively different dispersal processes, i.e. the Brownian motion (‘Gaussian walk’), representing a thin-tailed distribution, and a Levy flight (‘Cauchy walk’), with its power law decay representing a fat-tailed distribution.

### 3.1. Gaussian diffusion

A one-dimensional Gaussian diffusion process is defined by the following dispersal function:

3.21 $$\rho_{G}(x,Dt)=\frac{e^{-x^{2}/2Dt}}{\sqrt{2\pi Dt}},$$

where *D* is the diffusion coefficient. If we start the analysis by considering first the behaviour of the dispersal function 〈*ρ*_G_(*x*,*βζ*)〉 in the small time regime, for *ζ* sufficiently small it is expected that the main contribution would come from the damped Gaussian term e^−*ζ*^*ρ*_G_(*x*,*βζ*), as the integral term in equation (3.13) is associated with multiple bout sequences. In order to address its contribution, we must look at values of *ζ* where the stochasticity of the bout and rest sequences start to become relevant, which typically occurs at ζ≳1. The dispersal function in this regime is given by equation (3.15), with the integral being written as

3.22 ∫01dy(y−ymax)nρG(x,βζy)=∑m=0n(nm)(−ymax)n−m2πβζ(x22βζ)m+1/2Γ(−2m+12,x22βζ),

where *Γ*(*k*,*z*) is the upper incomplete Gamma function. If we look first at the asymptotic spatial regime, then with the aid of the expansion for large real arguments (z→∞),

3.23 $$\Gamma (n,z)≃z^{n-1}\;e^{-z}\sum_{k=0}\frac{\Gamma (n)}{\Gamma (n-k)}z^{-k},$$

it is possible to conclude that, in this intermediary time regime, the original Gaussian process is always asymptotically dominant because the leading order of every term defined by equation (3.22) presents a faster decay for large spatial values due to the presence of an *x*^−2^ factor. In a more formal way, the complete sum of the series expansion (3.15) for the Gaussian dispersal behaves asymptotically as

**Figure 2.**
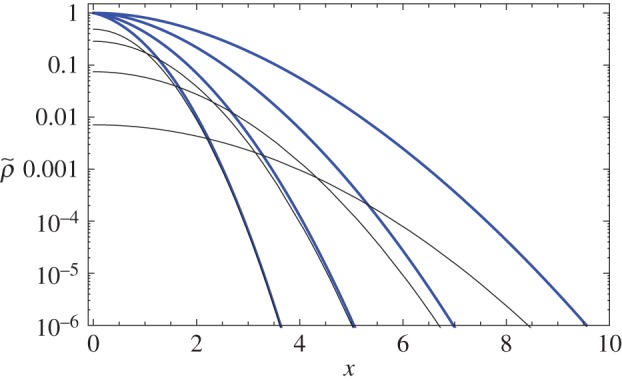
Rescaled dispersal functions for the Gaussian process at small time regime, for *β*=1 and *α*=2. From left to right, *ζ*=0.5,1,2,4. With the scaling factor *S*(*ζ*)=〈*ρ*_G_(0,*ζ*)〉, blue (thick) curves represent the rescaled dispersal $\left\langle {\tilde{\rho }}_{G}(x,\zeta )\rangle =S^{-1}\langle \rho_{G}(x,\zeta )\right\rangle$, and black (thin) curves the rescaled damped Gaussian *S*^−1^ *e*^−*ζ*^*ρ*_G_(*x*,*ζ*). The tail of the distribution is always determined by the tail of the original process, but as the system evolves its contribution becomes less significant, mostly restricted to large distances.

3.24 $$\sum_{n=0}^{\infty }a_{n}(\alpha ,\zeta )\int_{0}^{1}dy{\left(y-y_{\text{max}}\right)}^{n}\rho_{G}(x,\beta \zeta y)≃\sqrt{\frac{2\beta \zeta }{\pi }}K(\alpha ,\zeta ,1)\frac{e^{-x^{2}/2\beta \zeta }}{x^{2}},$$

which decays faster for large *x* than *e*^−*x*^2^/2*βζ*^ present in the original process. As a result, even though for small times the original Gaussian diffusion accounts for most of the dispersal curve, the exponential dumping diminishes its contribution as the system evolves, which leads to a restructure of the dispersal function that happens at small spatial scales and tends to spread to large ones as the time grows. For any fixed time, the bout and rest sequences contribute to a reshaping of the dispersal function for small *x* while keeping the tail of the original Gaussian process for large *x*: but, the larger the time *ζ* we look at the function, then we need to look at bigger values of *x* in order to have its tail described by the original process. So the picture we get at these intermediary times is one in which a slower spreading dispersal function 〈*ρ*_G_(*x*,*βζ*)〉, slower because of the presence of rest periods, is associated with fatter tails coming from the original faster dispersal *ρ*_G_(*x*,*βζ*), as shown in figure 2.

To understand what happens to the dispersal function at large times, we must resort to the Laplace method (3.20) to estimate the leading asymptotics. In this regime, the damped Gaussian term should not contribute any more, and the dispersal function may be approximated only by the asymptotic contribution of integral (3.17), resulting in

3.25 $$\langle \rho_{G}(x,\beta \zeta )\rangle =e^{-\zeta }\rho_{G}(x,\beta \zeta )+I_{G}(\alpha ,\zeta )≃\sqrt{\frac{1+\alpha }{2\pi \alpha \beta \zeta }}\;e^{-(1/2)((1+\alpha )/\alpha \beta \zeta )x^{2}}.$$

From equation (3.25), it is straightforward to conclude that asymptotically we obtain the following rescaling of the diffusion constant,

3.26 $$D^{*}(\alpha )=\frac{\alpha }{1+\alpha }D=\frac{\omega }{\omega +\lambda }D,$$

which coincides with the results of the semi-intuitive analysis in §2. Correspondingly, it leads to a renormalized diffusion process being described by

3.27 $$\langle \rho_{G}(x,Dt)\rangle ≃\rho_{G}(x,D^{*}t)=\frac{1}{\sqrt{2\pi D^{*}t}}\;e^{-x^{2}/2D^{*}t}.$$

**Figure 3.**
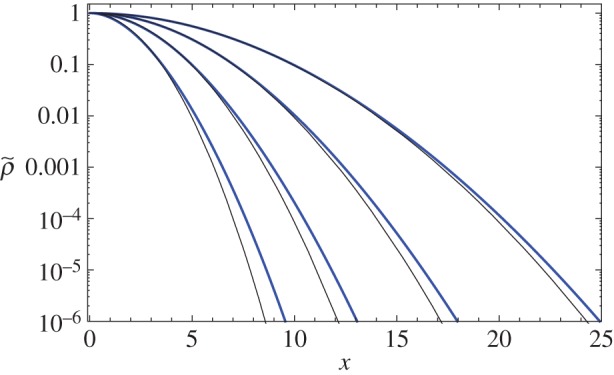
Rescaled dispersal functions for the Gaussian process at large-time regimes, for *β*=1 and *α*=2. From left to right, *ζ*=4,8,16,32. Blue (thick) curves represent the rescaled dispersal $\left\langle {\tilde{\rho }}_{G}(x,\zeta )\rangle ={\left\langle \rho_{G}(0,\zeta )\right\rangle }^{-1}\langle \rho_{G}(x,\zeta )\right\rangle$, while black (thin) ones represent the rescaled asymptotic diffusive behaviour *ρ*_G_(0,β*ζ)^−1^*ρ*_G_(*x*,β*ζ), as defined by equation (3.27), where β*=*βα*(1+*α*)^−1^. As the dispersal function converges to the asymptotic diffusion from above (at sufficiently large spatial values), for finite times the core of the function may be approximated by a Gaussian diffusion, but it still presents a faster than normal decay at the tails.

As the time evolution continuously transforms the dispersal function from the original *ρ*_G_(*x*,*Dt*) process into a slower dispersing process defined by *ρ*_G_(*x*,D*t), because *D*>D*, it also becomes evident why we observe a dispersal function with a somewhat fatter tail: whenever we combine the functional forms of two Gaussian processes characterized by different diffusivities, the result is not exactly a Gaussian process but rather a dispersal process characterized by the slower diffusion at small spatial scales, with fatter tails (from the faster diffusion) present at large spatial scales. Figure 3 shows the dispersal function with fatter than Gaussian tails, and how the convergence takes place for large times, thus confirming our conclusions.

In the case that the baseline process (non-interrupted movement) is a Brownian motion, so that the pdf 〈*ρ*(*x*,⋅)〉 is thin-tailed, a convenient alternative way to describe dispersal is to consider the MSD, i.e. the second moment of the pdf. It is well known that a non-interrupted diffusive process (i.e. bouts only, no rests) results in the MSD depending linearly on time, 〈*x*^2^〉∼*Dt*, where *D* is the diffusion coefficient. Now we are going to consider the MSD for the composite movement combining alternating bouts and rests in order to reveal how this linear dependence can be modified.

From the definition of the MSD for the dispersal process, we obtain

3.28 $$\begin{array}{rl} \left\langle x^{2}(\alpha ,\beta ,\zeta )\right\rangle & \equiv \int_{-\infty }^{\infty }dxx^{2}\langle \rho_{G}(x,\beta \zeta )\rangle \\ & =\beta \zeta \left\{e^{-\zeta }+\zeta \;e^{-\alpha \zeta }\int_{0}^{1}dy\;e^{(\alpha -1)\zeta y}y\left[\sqrt{\frac{\alpha y}{1-y}}I_{-1}(h)+I_{0}(h)\right]\right\}. \end{array}$$

Based on our above analysis of small-time and large-time asymptotics, it is readily seen that

3.29 $$\langle x^{2}(\alpha ,\beta ,\zeta )\rangle ∼\beta \zeta \;\text{for}\;\zeta \ll 1\;\text{and}\;\langle x^{2}(\alpha ,\beta ,\zeta )\rangle ∼\beta^{*}\zeta \;\text{for}\;\zeta \gg 1,$$

where β*=*βα*/(1+*α*).

**Figure 4.**
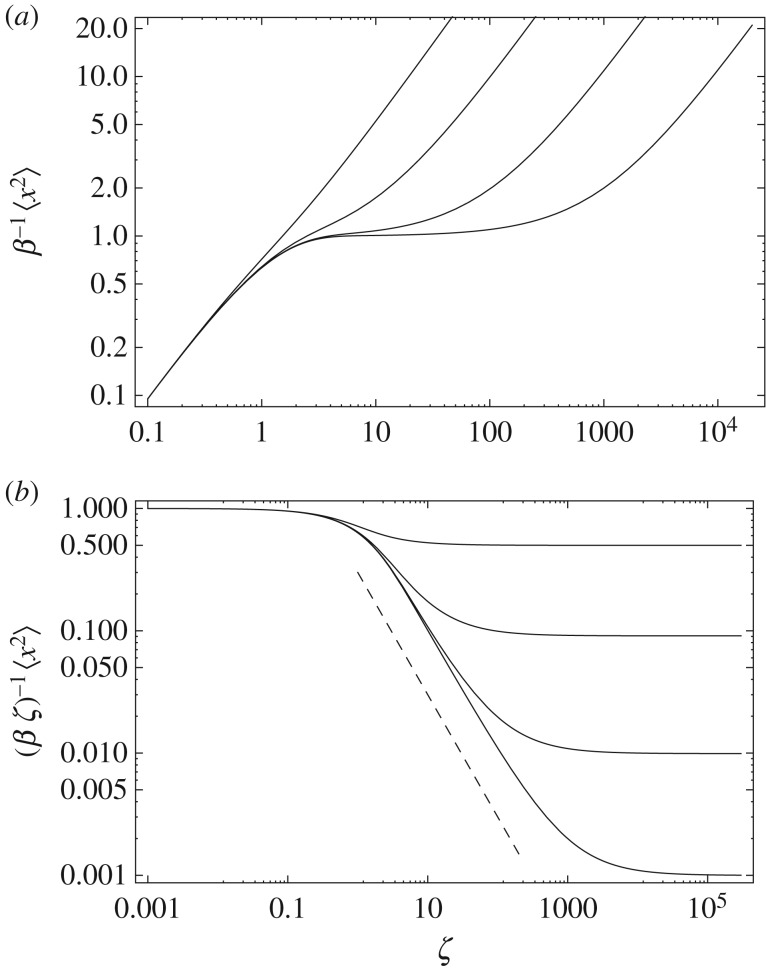
Mean squared displacement of the dispersal process, (*a*) in the original variables, (*b*) in rescaled variables. In both panels, curves are shown for (from bottom to top) *α*=10^−3^,10^−2^,10^−1^ and 10^0^. The dashed line shows a power law proportional to *ζ*^−1^. When the expected value of the rest periods is much larger than the bout ones (*α*≪1), the system exhibits long periods of almost no movement, which accounts for the 〈*x*^2^〉≈*const*. If *α* is increased until the expected periods become comparable (*α*∼1), then the MSD presents a power law type of transient behaviour of the form 〈*x*^2^〉∼*ζ*^*γ*^, with *γ*∈(0,1), which is characteristic from a subdiffusive type of spread.

**Figure 5.**
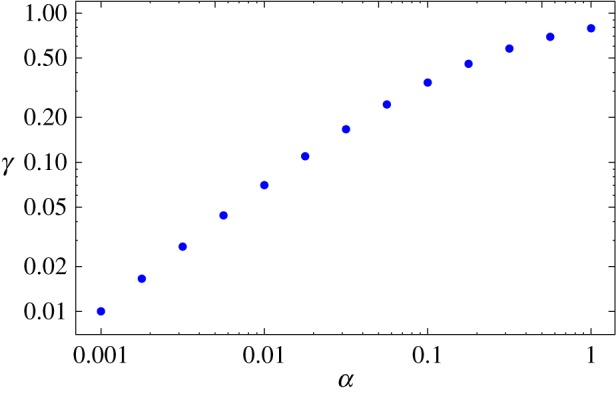
Exponent of the transient subdiffusive spread 〈*x*^2^〉∼*ζ*^*γ*^ as a function of the ratio *α* of expected periods of movement and rest. The exponent *γ* is calculated as the maximum slope of 〈*x*^2^〉(*t*), cf. figure 4.

Comparing the small-time and large-time asymptotics in (3.29), intuitively, one can expect that, at intermediate time, the movement slows down. Unfortunately, for the intermediate asymptotics *ζ*∼1, analytical investigation does not seem to be possible because of the complexity of the corresponding equation (3.15). The dynamics on the intermediate time scale is therefore studied through Monte-Carlo simulations. The results are shown in figure 4. It appears that, on the intermediate time scale which can span over a few orders of magnitude, the movement shows clear subdiffusive properties so that 〈*x*^2^〉∼*t*^*γ*(*α*)^, where the exponent *γ* can be anywhere between 0 and 1, being an increasing function of *α* (figure 5). The exponent tends to zero as α→0, because the system tends to stay in rest for most of the time, and γ→1 as α→∞, because of the opposite situation where the animal keeps moving almost all the time as the proportion of bout duration becomes much greater than the proportion of rests. Interestingly, if this result is considered together with the properties of the dispersal kernel, the movement has somewhat counter-intuitive properties as the subdiffusive spread occurs for the kernel with the tail fatter than Gaussian.

### 3.2. Levy flights

As an example of a fat-tailed ‘superdiffusing’ process, we are going to consider the Cauchy walk that is often regarded as a standard model of Levy flights [12]. In the one-dimensional case, the corresponding dispersal kernel is given by the following expression:

3.30 $$\rho_{C}(x,Dt)=\frac{1}{\pi }\frac{Dt}{x^{2}+{\left(Dt\right)}^{2}}=\frac{1}{\pi }\frac{\beta \zeta }{x^{2}+{\left(\beta \zeta \right)}^{2}}.$$

To describe the behaviour of the dispersal process in intermediary times, we must consider the expansion (3.15), with the integral being written as

3.31 ∫01dy(y−ymax)nρC(x,βζy)=∑m=0n(nm)(−ymax)n−mβζ(n+2)πx22F1(1,n2+1;n2+2;−β2ζ2x2),

where _2_*F*_1_(*a*,*b*;*c*;*z*) is the hypergeometric function, as defined by

3.32 $${}_{2}F_{1}(a,b;c;z)=\sum_{n=0}^{\infty }\frac{{\left(a\right)}_{n}{\left(b\right)}_{n}}{{\left(c\right)}_{n}}\frac{z^{n}}{n!}=\frac{\Gamma (c)}{\Gamma (b)\Gamma (c-b)}\int_{0}^{1}dyy^{b-1}{\left(1-y\right)}^{c-b-1}{\left(1-zy\right)}^{-a},$$

and (*q*)_*n*_ is the Pochhammer symbol representing the rising factorial,

3.33 $${\left(q\right)}_{n}=\frac{\Gamma (q+n)}{\Gamma (q)}.$$

Differently from the Gaussian process, where we were able to show that the presence of bout and rest periods tend to affect the dispersal kernel in a way that propagates from the origin (bulk) towards the tails, the effect on the Cauchy dispersal appears in a global way, because the perturbations induced are of the same order as the dispersal process itself, as may be observed from the spatially asymptotic contribution of the integral term, i.e.

3.34 $$\sum_{n=0}^{\infty }a_{n}(\alpha ,\zeta )\int_{0}^{1}dy{\left(y-y_{\text{max}}\right)}^{n}\rho_{C}(x,\beta \zeta y)≃\frac{\beta \zeta }{\pi x^{2}}\sum_{n=0}^{\infty }\frac{{\left(1-y_{\text{max}}\right)}^{n}}{n+2}a_{n}(\alpha ,\zeta ).$$

Even though we are observing a process where the dispersal kernel is being restructured across all spatial scales simultaneously (which is probably related to the fact that the dispersal function is also scale-free), the dispersal behaviour at large times shows the same general property as the Gaussian process, as asymptotically we also recover a renormalized Cauchy type of dispersal defined by

3.35 $$\langle \rho_{C}(x,\beta \zeta )\rangle ≃\rho_{C}(x,\beta^{*}\zeta )=\frac{1}{\pi }\frac{\beta^{*}\zeta }{x^{2}+{\left(\beta^{*}\zeta \right)}^{2}},$$

where the scaling relation for the distribution parameter, that is

3.36 $$\beta^{*}=\frac{\alpha }{1+\alpha }\beta ,$$

is exactly the same as in the case of the Brownian motion.

### 3.3. Universality of rescaling

From the analysis of the two above-mentioned examples of dispersal processes, one cannot leave unnoted the emerging behaviour at large-time regimes, as both dispersal functions recover their initial functional forms in a renormalized time scale. In fact, if the observation time *ζ* is sufficiently large, it should be expected that the fluctuations arising from the random sampling tends to disappear, as the system would settle into a regime where it splits its whole activity into well-defined portions of rest and bout periods. If this statement is indeed true, then the rescaling feature should be dependent only on the statistical nature of the activity periods sampling, thus being observable with any dispersal function at hand. Although we cannot formally prove this conjecture, we can show some evidence strongly supporting the statement if we look at the expected duration of the total amount of bout period that takes place within a given observation time *ζ*, which may be written as

3.37 $$\langle \Delta t_{b}(\zeta )\rangle =\zeta \;e^{-\zeta }+\zeta^{2}\;e^{-\alpha \zeta }\int_{0}^{1}dy\;e^{(\alpha -1)\zeta y}y\left[\sqrt{\frac{\alpha y}{1-y}}I_{-1}(h)+I_{0}(h)\right].$$

For *ζ* sufficiently large, having applied the Laplace method, one can show that this function tends to a linear time evolution that, when written in the form

3.38 $$\frac{\left\langle \Delta t_{b}(\zeta )\right\rangle }{\zeta }≃\frac{\alpha }{1+\alpha },$$

enables us to interpret the asymptotic dynamics as a constant fraction of the movement spent on bout activity. Hence, any type of dispersal function that has its proper behaviour altered by the statistical distribution of bout and rest periods should be described by its original function with this rescaled time, and if the degree of spatial dispersal of the function at a given time *ζ* is characterized by a single constant entering the functional form only as a time multiplier, then the whole change in behaviour should be characterized by the renormalization process

3.39 $$\beta \zeta \to \beta^{*}(\alpha )\zeta ,$$

independent of the specifics of the function.

## 4. Convergence rates

As was shown above, the dispersal process 〈*ρ*_G_(*x*,*βζ*)〉 converges to the asymptotic diffusion *ρ*_G_(*x*,β*ζ). However, details of the convergence have remained unclear. One way of approaching this problem is to define a measure of how different the two functions are and look for general properties of its time evolution. With that in mind, we are going to define a global convergence function (or global error function) as

4.1 $$R(\alpha ,\beta ,\zeta )\equiv \int_{-\infty }^{\infty }dx{\left[\langle \rho (x,\beta \zeta )\rangle -\rho (x,\beta^{*}\zeta )\right]}^{2},$$

which gives a measure of the proximity of the two functional forms at a given time *ζ*, and enable us to determine via an asymptotic analysis the typical time scale of the convergence. But there is a scaling issue with this function, because the divergence appearing as ζ→0 does not allow one to define a proper (natural) scale for comparison. To circumvent this problem, we may consider a scaling function defined by the integrated squared difference between two arbitrary Gaussian processes, i.e.

4.2 $$\Lambda_{G}(\beta_{1},\beta_{2},\zeta )\equiv \int_{-\infty }^{\infty }dx{\left[\rho_{G}(x,\beta_{1}\zeta )-\rho_{G}(x,\beta_{2}\zeta )\right]}^{2}=\frac{1}{2\sqrt{\pi }}\left[\frac{1}{\sqrt{\beta_{1}}}+\frac{1}{\sqrt{\beta_{2}}}-\frac{2\sqrt{2}}{\sqrt{\beta_{1}+\beta_{2}}}\right]\frac{1}{\zeta^{1/2}}.$$

**Figure 6.**
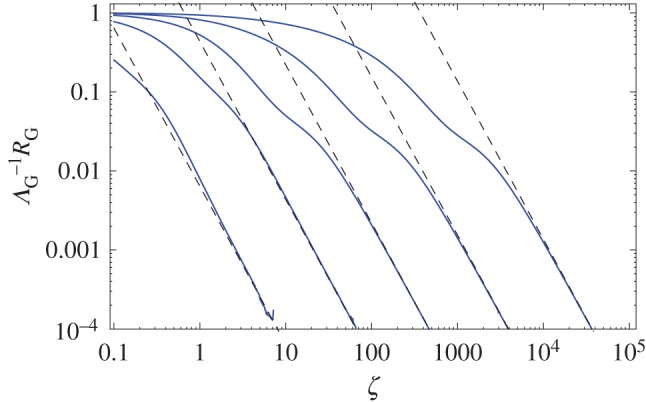
Ratio between the global convergence (*R*_*G*_) and maximum difference (*Λ*_*G*_) functions for the Gaussian dispersal. From left to right, *α*=10^*n*^, with *n*= 1,0,−1,−2,−3. Continuous curves (blue) are the numerical evaluation of the ratio function, while dashed (black) lines represent the asymptotic behaviour as defined by equation (4.7).

As this scaling function presents the same divergence when two distinct diffusivities (*β*_1_ and *β*_2_) are considered, we may look at the ratio between the global convergence function *R*(*α*,*β*,*ζ*) and the maximum possible difference between 〈*ρ*(*x*,*βζ*)〉 and *ρ*(*x*,β*ζ), as defined by *Λ*_*G*_(*β*,β*,*ζ*), which gives a natural way of measuring the time evolution of global functional distances because the ratio is always defined on the interval (0,1). If we perform a numerical evaluation (figure 6), then it is possible to observe that intermediate times are characterized by a non-uniform evolution of the ratio, but asymptotically the convergence presents a well-defined power law type of convergence. As our main interest lies in determining the typical behaviour on this asymptotic limit, we must look for a way to determine higher order contributions coming from integrals of the type (3.18), but this involves a re-examination of the Laplace method.

When considering integrals of the type (3.18), we typically consider a series expansion around the maximum *y*_0_ of the function *S*(*y*), followed by a Gaussian type of approximation of the integral, which is only justified if the kernel is sufficiently sharp around its neighbourhood. Because we are interested in determining the first few orders of contribution in the asymptotic limit ζ→∞, let us consider the following expansion:

4.3 $$\begin{array}{rl} I(\zeta ) & =\int_{0}^{1}dy\left(f_{0}+f_{0}^{\prime }z+f_{0}^{\prime \prime }\frac{z^{2}}{2}+\cdots \right)e^{\zeta [S_{0}+S_{0}^{\prime }z+S_{0}^{\prime \prime }(z^{2}/2)+S_{0}^{\left(3\right)}(z^{3}/3!)+S_{0}^{\left(4\right)}(z^{4}/4!)+\cdots ]}\\ & \approx e^{\zeta S_{0}}\int_{-\infty }^{\infty }dz\;e^{\zeta S_{0}^{\prime \prime }(z^{2}/2)}\left(f_{0}+f_{0}^{\prime }z+f_{0}^{\prime \prime }\frac{z^{2}}{2}+\cdots \right)\sum_{n=0}^{\infty }\frac{\zeta^{n}{\left[S_{0}^{\left(3\right)}(z^{3}/3!)+S_{0}^{\left(4\right)}(z^{4}/4!)+\cdots \right]}^{n}}{n!}, \end{array}$$

where the notation used is defined by

4.4 $$z=y-y_{0},\;f_{0}^{\left(n\right)}=\partial_{y}^{n}f(y,\zeta ){|}_{y=y_{0}},\;S_{0}^{\left(n\right)}=S^{\left(n\right)}(y){|}_{y=y_{0}}.$$

Now, if the derivatives $f_{0}^{\left(n\right)}$ do not show an increase in the order in *ζ* when compared with the leading order term *f*_0_, then the next leading order will come from terms whose expansion in *ζ*^*δ*_1_^*z*^*δ*_2_^ satisfy *δ*_2_=2(*δ*_1_+1), because each integration of *z*^2*n*^ generates a *ζ*^−(*n*+1/2)^ multiplying factor. When taking this into account, it is possible to conclude that the second leading order will come from contributions of the following approximation:

4.5 $$I(\zeta )\approx e^{\zeta S_{0}}\int_{-\infty }^{\infty }dz\;e^{\zeta S_{0}^{\prime \prime }\frac{z^{2}}{2}}\left[f_{0}+f_{0}^{\prime \prime }\frac{z^{2}}{2}+\left(\frac{f_{0}S_{0}^{\left(4\right)}}{4!}+\frac{f_{0}^{\prime }S_{0}^{\left(3\right)}}{3!}\right)\zeta z^{4}+\frac{f_{0}}{2}{\left(\frac{S_{0}^{\left(3\right)}}{3!}\right)}^{2}\zeta^{2}z^{6}\right].$$

If we come back now to the specific case of the Gaussian dispersal, and remember to take into account the second term of the asymptotic Bessel expansion (3.16), then it is possible to compute the asymptotic behaviour of the convergence function as

4.6 $$R_{G}(\alpha ,\beta ,\zeta )≃\frac{33}{512{\left(\pi \beta \right)}^{1/2}\alpha^{5/2}{\left(1+\alpha \right)}^{3/2}}\frac{1}{\zeta^{5/2}},$$

thus leading to the asymptotic ratio

4.7 $$\frac{R_{G}(\alpha ,\beta ,\zeta )}{\Lambda_{G}(\beta ,\beta^{*},\zeta )}≃\frac{33}{256{\left[\alpha (1+\alpha )\right]}^{3/2}}{\left[\sqrt{\alpha (1+\alpha )}+\alpha \left(1-2\sqrt{\frac{2(1+\alpha )}{1+2\alpha }}\right)\right]}^{-1}\frac{1}{\zeta^{2}},$$

which matches perfectly the numerical results (figure 6). Our result shows that the convergence time asymptotically decays proportionally to *ζ*^−2^, so in order to obtain a typical time scale of convergence one needs only to define an arbitrarily small value *ϵ* of comparison for the ratio and invert equation (4.7) to determine the time *ζ*(*ϵ*) required for the system to reach that confidence level.

**Figure 7.**
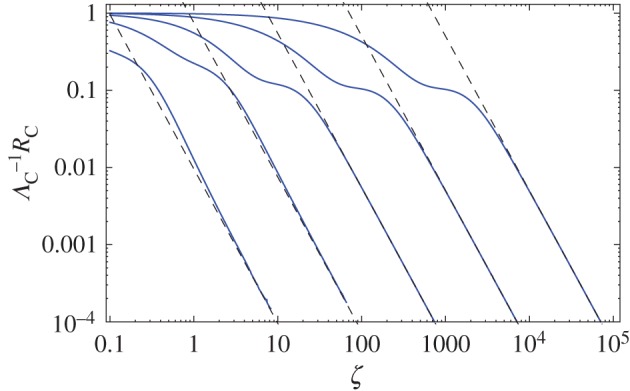
Ratio between the global convergence (*R*_*C*_) and maximum difference (*Λ*_*C*_) functions for the Cauchy type of dispersal. From left to right, *α*=10^*n*^, with *n*=1,0,−1,−2,−3. Continuous curves (blue) are the numerical evaluation of the ratio function, while dashed (black) lines represent the asymptotic behaviour as defined by equation (4.8).

In the case of the Cauchy walk, as this dispersal also shows a convergence between two functional forms, it is possible to use the same convergence function (4.1) to determine the typical time scale of convergence for the Cauchy dispersal. The numerical evaluation of the ratio function is shown in figure 7, and its asymptotic behaviour may be obtained, after some calculations, as

4.8 $$\frac{R_{C}(\alpha ,\beta ,\zeta )}{\Lambda_{C}(\beta ,\beta^{*},\zeta )}≃\frac{1+2\alpha }{2\alpha^{2}(1+\alpha )}\frac{1}{\zeta^{2}},$$

which clearly shows the same type of time scaling *ζ*^−2^ as the Gaussian dispersal.

## 5. Discussion and concluding remarks

In this paper, we considered the properties of the composite random walk consisting of alternating periods of movement (bouts) and rest. Having used the time scale separation argument, i.e. that the periods of rest between the bouts occur on a larger time scale compared with possible short stops of the baseline ‘uninterrupted’ random movement, we performed a rigorous mathematical analysis of this composite walk. Namely, we calculated the ‘expected’ probability density function ⟨ρ(x,Dt)⟩ of the position of the walker which provides a global description of the composite movement across different time scales and over different stochastic realizations of bouts and rests. This global pdf 〈*ρ*〉 appears to be related to the probability density function ρ(x,Dt) of the corresponding uninterrupted movement. In a general case, the pdf of the composite movement is given by the integral relation (3.11). We then focused on two specific cases where the pdf of the uninterrupted movement is (i) a normal distribution quantified by the diffusion coefficient and (ii) the Cauchy distribution quantified by its parameter. For each of these two cases, we obtained the explicit expressions for the probability distribution of the composite walk in the large-time asymptotics, see equations (3.27) and (3.35), respectively.

With regard to the questions itemized in the introduction, we therefore have obtained the following answers:

— Regardless of the properties of the uninterrupted movement, i.e. whether it is the Brownian motion described by (3.21) or the Levy walk described by (3.30), the evolution of the pdf in time follows the same scenario resulting, in the large-time asymptotics, in the rescaling of the distribution parameter in the same way, cf. equations (3.26) and (3.36). The functional form of the composite walk’s pdf therefore coincides with the pdf of the uninterrupted movement subject to the ‘universal’ rescaling of the distribution parameter; see (3.39). For the intermediate time scale, the pdf of the composite movement is given by the integral expression (3.11) which, as we have shown by numerical simulations, predicts a somewhat fatter tail compared with the pdf of the uninterrupted movement (figure 3).
— In the case that the ‘baseline’ uninterrupted movement is described by the Brownian motion, on the intermediate time scale the MSD shows clear subdiffusive properties, i.e. 〈*x*^2^〉∼*t*^*γ*^ with 0<*γ*<1, where the value of the exponent *γ* depends on the ratio of the mean bout duration and the mean rest time.

Note that the analysis done in §3.3 suggests that the rescaling (3.39) should remain the same regardless the properties of the dispersal kernel. Regarding the convergence rate in the large-time asymptotics (see §4), although we cannot prove universality in a general case, the fact that the rate of convergence is the same (i.e. ∼*ζ*^−2^) in the two very different cases such as the Brownian motion and the Cauchy walk suggests that this is a general result too.

The subdiffusive movement on the intermediate time scale is a somewhat surprising result. We mention here that subdiffusion is widely observed in physical and chemical systems [31], where it usually appears as a result of particle trapping by certain spatial structures of the system (referred to as ‘traps’). By contrast, in our study the animal moves in a perfectly uniform environment without traps or refuges. The periods of immobility (i.e. rest) are attributed to purely behavioural reasons. Indeed, the existence of behaviour is a factor that can make movement of animals significantly different from movement of inanimate particles, cf. [26].

An interesting question is how the composite movement can be modified if the distribution of the rest times is not exponential but is described by a fat-tailed distribution (e.g. a power law). Although we cannot currently provide a quantitative study into this issue, the results that we obtained in this paper make it possible to figure out what the movement properties are going to be. A fatter-tailed distribution of the rest times would obviously result in further slowing down the movement on the intermediate scale, hence making the emerging subdiffusive behaviour more prominent. In the large-time asymptotics, however, one should expect that the functional form of the pdf of the uninterrupted movement is restored, subject to the rescaling of the corresponding distribution parameter as given by the universal relation (3.39).

We point out that our findings (such as the general expression (3.11) for the dispersal kernel and the universal rescaling (3.39) valid in the large-time asymptotics) have immediate ecological applications. Provided the statistical properties of the bouts and rests are known—which can be obtained from a small-scale experimental study—and the pattern of uninterrupted individual animal movement is established, it can be upscaled to the large-time ecological scale to give the dispersal kernel. Once the dispersal kernel is thus obtained, it can be used to construct kernel-based mathematical models describing the spatio-temporal dynamics of a given population over a broad range of spatial and temporal scales, e.g. see [4].

A question may arise here about the generality of the above message. Indeed, our results were obtained under some simplifying assumptions. For instance, our model of composite movement did not take into account the heterogeneity of the environment where the animal lives and moves; meanwhile, it is known that the complexity of the environment can affect the movement pattern [15,32]. However, here we argue that, unless the environment has a large-scale structure, e.g. as given by the presence of mountains or lakes, a small-scale heterogeneity is likely to affect only the choice of the movement step, which is already described by the pdf.

In conclusion, we mention that our study obviously leaves a few open problems. Firstly, we assumed that the animal uses only one movement mode, e.g. as described by a single probability distribution. In reality, however, animals often have more than one mode [19,33,34] and switching between the modes may depend on a variety of factors, in particular on the spatial and temporal scales of the movement [35]. Whether the existence of different movement modes may or may not affect the upscaling relation (4.1) is not immediately clear. Secondly, here we have considered only the individual movement, thus neglecting any interaction between the animals; however, the latter is known to affect their movement [36], for instance, through formation of groups such as schools, packs, herds, flocks, etc. [37]. Moreover, some recent results show that there may exist a more intricate collective behaviour such as density-dependent cut-off when the movement type changes at a certain spatial scale because of the interactions with conspecific animals [38]. Although in the first instance we can argue, similarly to the above, that the interactions with the other animals should already be taken into account by a properly chosen pdf of the uninterrupted movement, this issue requires a more careful consideration. All these issues should become a focus of the future study.

## Footnotes

1 In physical applications, the quantity of a similar meaning is called a propagator, e.g. see [6].

## Data accessibility

This study does not have any data.
